# Exploring the Feasibility of Daycase Pediatric Tonsillectomy in a Tertiary Care Hospital: A Retrospective Review

**DOI:** 10.7759/cureus.89560

**Published:** 2025-08-07

**Authors:** Rana S Alkatheeri, Mustafa A Abdul Latif, Shayan S Ansari

**Affiliations:** 1 Department of Otolaryngology, Tawam Hospital, Al Ain, ARE

**Keywords:** adenoidectomy, adenotonsillectomy, daycase, pediatric otolaryngology, tertiary care, tonsillectomy

## Abstract

Introduction

Tonsillectomy, with or without adenoidectomy, is one of the most commonly performed procedures in pediatric otolaryngology. Over the years, there has been a shift in postoperative practice from routine admissions to daycase procedures. The study aimed to evaluate the postoperative course of pediatric tonsillectomy with or without adenoidectomy in a local tertiary care hospital, and to compare our current practices with the internationally published data on the subject.

Methods

A retrospective chart review was conducted at Tawam Hospital in the UAE. We included pediatric patients aged one to 15 years who underwent tonsillectomy with or without adenoidectomy at our hospital. The study was conducted over a six-month period from December 1, 2023, to May 31, 2024.

Results

A total of 65 patients were included in the study. Thirty-nine patients (60%) were admitted postoperatively, while 26 (40%) were discharged the same day. When the UK guidelines were applied, the suggested admissions dropped to 31 (47.7%), and the daycase procedures increased to 34 (52.3%). Both of the daycase rates were lower than the 80% target recommended by the Getting It Right First Time (GIRFT) programme.

Conclusion

Pediatric adenotonsillectomy still appears to be a safe daycare procedure in a tertiary care hospital. However, achieving an 80% daycase rate might not be possible in our population due to patients’ specific factors.

## Introduction

Adenoidectomy and tonsillectomy are among the most common surgical procedures undertaken by pediatric patients under the age of 15 years. In the majority of cases, these procedures are being performed for symptoms of sleep-disordered breathing (SDB) or recurrent infections [1]. Traditionally, the postoperative course included an overnight admission to monitor for any desaturation or respiratory distress. However, there is a shift toward daycase adenotonsillectomy, while preserving inpatient monitoring for a selected group of patients [2]. In 2019, following the COVID-19 pandemic and its sequelae on health care systems, the Getting It Right First Time (GIRFT) programme was established. The UK national GIRFT report for ear, nose, and throat (ENT) surgery suggested a target of 80% daycase rate of tonsillectomy [3].

Study objectives

The primary objective of this study is to examine our local practice in a tertiary care hospital regarding pediatric tonsillectomy and adenotonsillectomy. We aim to assess its safety and feasibility in our patient population and compare our discharge rates with internationally accepted benchmarks.

## Materials and methods

A retrospective cohort study that analyzed data retrieved from Cerner (Cerner Corporation, Kansas City, MO) electronic health records of pediatric patients who underwent tonsillectomy with or without adenoidectomy at Tawam Hospital over a six-month period. The patients were operated on by multiple surgeons at the department, each using their preferred surgical techniques. The study investigated patients' specific factors, including age, gender, weight, nationality, distance from hospital, patient’s past medical history, including previous hospital admissions, history of SDB and apneas, their anticipated postoperative disposition, and the actual final disposition, and finally any documented postoperative complications.

Data collection

Data were collected from Cerner electronic health records, known as “Salamtak.” Pediatric patients aged one to 15 years who underwent tonsillectomy with or without adenoidectomy during the six-month period from December 1, 2023 to May 31, 2024 were included. Sixty-five patients were accepted into the study based on the inclusion and exclusion criteria. Demographic data, medical comorbidities, previous admissions, history of SDB and observed apneas, type of operation, and postoperative complications were collected and encrypted into non-identifiable data. 

Inclusion and exclusion criteria 

We included all pediatric patients aged one to 15 years who underwent tonsillectomy, with or without adenoidectomy, during the six-month period described earlier. Patients who underwent myringotomy with or without grommet insertion, along with adenotonsillectomy, were also included. Multiple tonsillectomy surgical techniques were included. The included cases were performed by multiple ENT surgeons in our hospital. We excluded cases that lacked proper electronic documentation about the postoperative disposition or complications data. We also excluded patients who had concurrent major surgery performed at the same time. 

Data analysis

Data were analyzed using SPSS (IBM SPSS Statistics for Windows, IBM Corp., Version 26, Armonk, NY). Continuous variables (age, BMI centile) were categorized and tested against daycase and admission status. All other variables were also tested against daycase and admission status. All of the data were compared using the chi-square test. A p-value ≤ 0.05 was considered statistically significant.

## Results

Demographics

**Figure 1:**
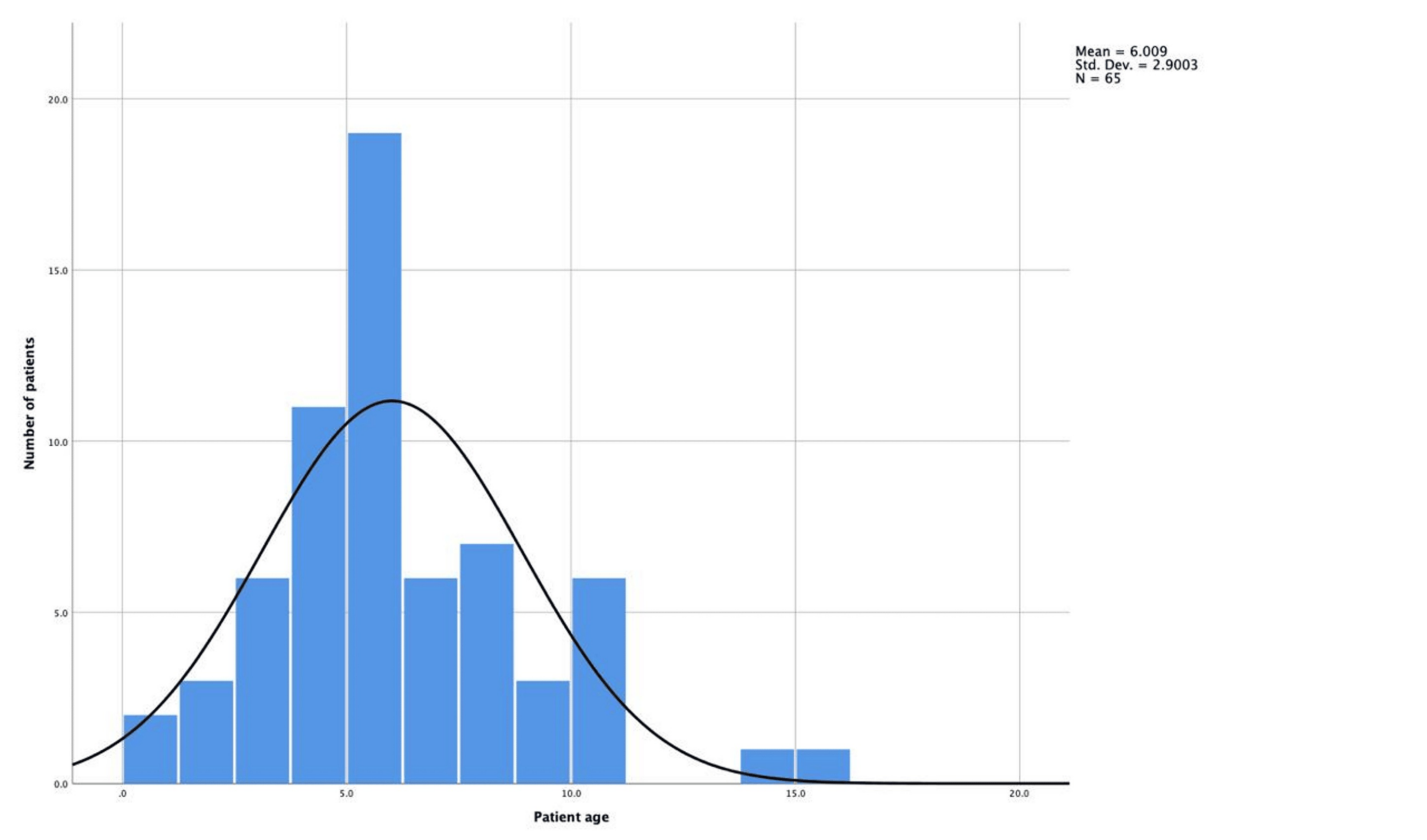
Age distribution of adenotonsillectomy patients The histogram shows the frequency of patients (y-axis) across different age groups in years (x-axis). A total of 65 patients are included. The black curve represents a normal distribution fitted to the data. The mean age is 6.01 years with a standard deviation of 2.9 years.

A total of 65 pediatric patients underwent tonsillectomy with or without adenoidectomy during the study period. The mean age was 6.01 ± 2.9 years (Figure 1), and the mean weight was 30.17 ± 20.57 kg. Among the patients, 39 (60%) were male, and 26 (40%) were female (Table 1).

**Table 1:** Patients’ demographics in admission and daycase groups The total number of patients in the admission and daycase groups is shown as n (number of patients). Percentages reflect the proportion within each group.

Patient demographics	Admission (n = 39; 60%)	Daycase (n = 26; 40%)
Age (mean ± SD in years)	6.01 ± 2.9 years	
Gender	Males: 22 (56.4%)	Males: 17 (65.4%)
	Females: 17 (43.6%)	Females: 9 (34.6%)
Nationality	Locals: 34 (87.2%)	Locals: 17 (65.4%)
	Non-locals: 5 (12.8%)	Non-locals: 9 (34.6%)

BMI-for-age

**Figure 2:**
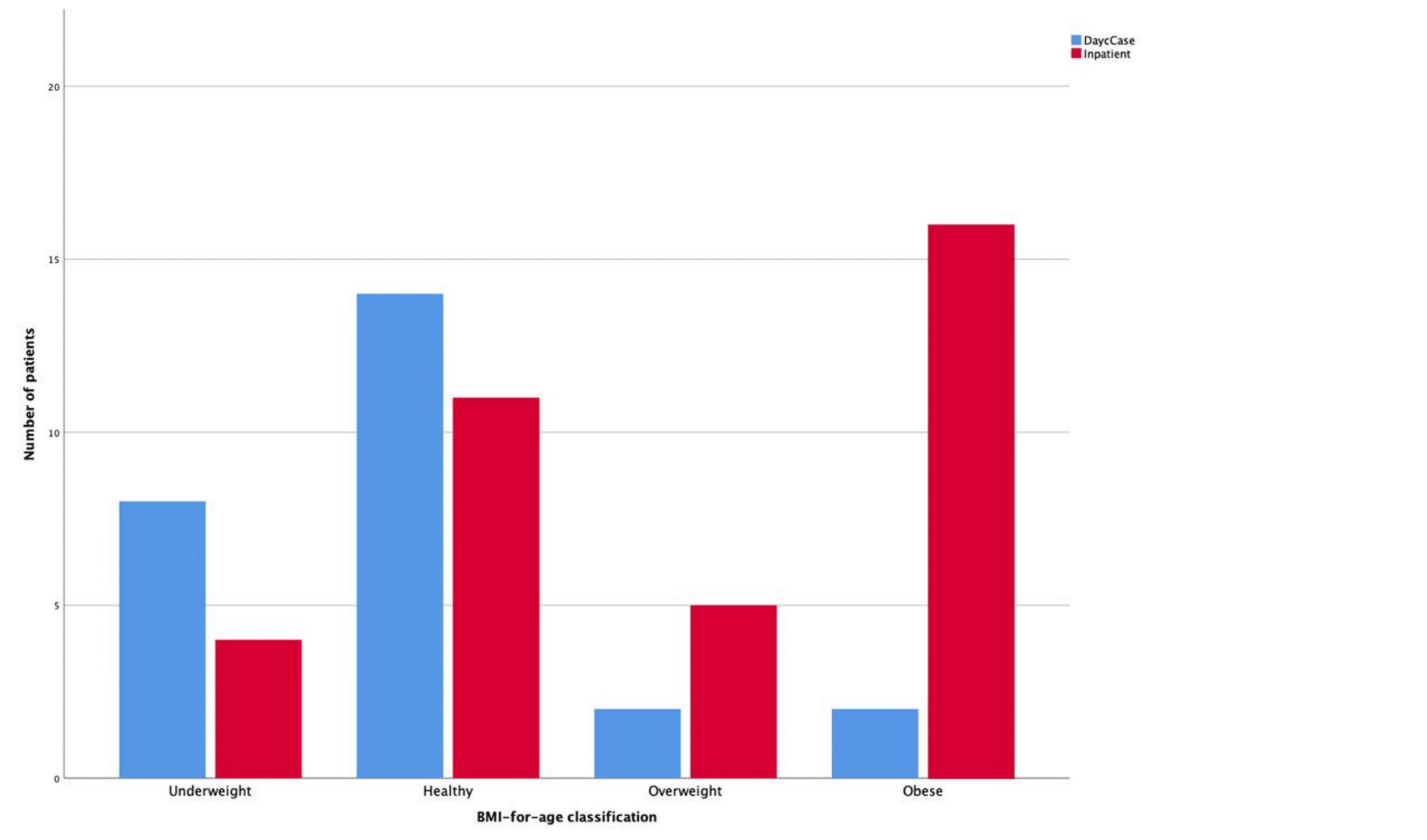
Comparison of inpatient admissions and daycase procedures across different BMI-for-age categories Distribution of patients by BMI-for-age classification stratified by Daycase and Inpatient status. Data are presented as number of patients (n) within each category.

BMI-for-age centiles were recorded for patients ≥2 years old (N = 62, 95.4%). The distribution was as follows: 18 (29.0%) obese, seven (11.3%) overweight, 25 (40.3%) healthy weight, and 12 (19.4%) underweight. Most obese (n = 16, 88.9%) and overweight (n = 5, 71.4%) patients were admitted, while the majority of healthy (n = 14, 56%) and underweight (n = 8, 66.7%) patients underwent daycase surgery (Figure 2). Distribution of BMI-for-age classifications among admitted (n = 36) and daycase (n = 26) patients aged ≥2 years is demonstrated in detail in Table 2. A chi-square test revealed a statistically significant difference in the distribution of BMI-for-age classifications between admitted and daycase patients (p = 0.006).

**Table 2:** BMI-for-age classification for patients ≥2 years Distribution of BMI-for-age classifications among admitted patients (n = 36) and daycase patients (n = 26) over two years of age (total N = 62). Data are presented as number of patients (n) and percentages (%). A p-value < 0.05 was considered statistically significant.

BMI-for-age classification for patients ≥2 years (N = 62)	Admission (n = 36)	Daycase (n = 26)	Chi-square (p-value)
Underweight	4 (11.1%)	8 (30.8%)	p = 0.006
Healthy	11 (30.6%)	14 (53.8%)	
Overweight	5 (13.9%)	2 (7.7%)	
Obese	16 (44.4%)	2 (7.7%)	

Surgical details and disposition 

Of the cohort, four patients underwent tonsillectomy without adenoidectomy (6.2%). Out of all tonsillectomies, 11 (16.9%) procedures were performed as intracapsular/reduction tonsillectomies. Myringotomy was performed in 22 patients (33.8%), with 20 (30.8%) requiring grommet insertion. Daycase surgery was achieved in 26 patients (40%), while 39 (60%) required inpatient admission, including one planned postoperative PICU admission (1.5%).

Medical comorbidities and previous admissions

**Figure 3:**
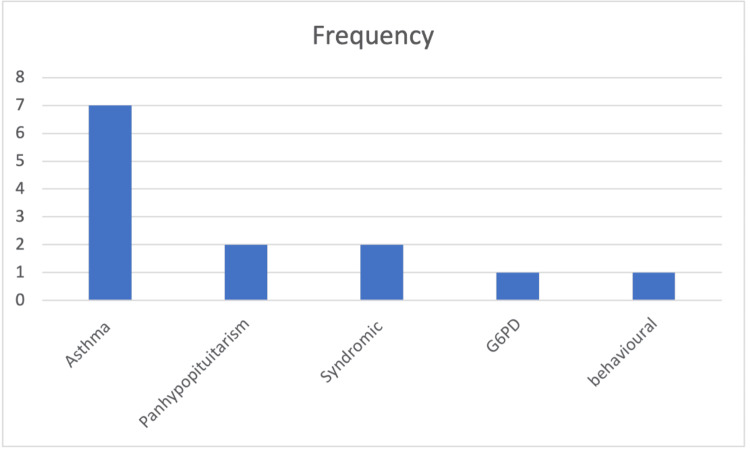
Medical comorbidities in the study population Frequency of medical comorbidities among admitted patients (N = 11). Asthma was the most common (n = 7; 63.6%), followed by panhypopituitarism and syndromic conditions (n = 2 each; 18.2%). Glucose-6-phosphate dehydrogenase (G6PD) deficiency and behavioral disorders were each reported in one patient (9.1%).

Medical comorbidities were present in 11 (28.2%) of the admitted patients, compared to none in the daycase group, a statistically significant difference (p = 0.003). A history of prior hospital admission was found in 13 (37.1%) of admitted patients and three (12%) of the daycase group (p = 0.03). Medical comorbidities found in our study population are listed in Figure 3. It is worth noting that some patients had more than one medical condition at the same time, necessitating hospital admission.

Sleep-disordered breathing and sleep apnea

A history suggestive of SDB with observed sleep apnea was present in 41 patients (63.1%), 28 (68.3%) of them were in the inpatient category, and only 13 (31.7%) were from daycase (Table 3). The p-value calculated using the chi-square test was p = 0.074. Although this indicates that the result was not statistically significant, the higher number of inpatient procedures among patients with sleep apnea may represent a trend that could become more apparent with a larger sample size.

**Table 3:** Distribution of sleep-disordered breathing (SDB) history by admission type Distribution of SDB with observed sleep apnea among inpatient and daycase patients. Data are presented as number of patients (n/N) and corresponding percentages, where N refers to the total number of patients in each category. A chi-square test was used to assess statistical significance (p = 0.074).

Sleep apnea status	Inpatient (n = 39)	Daycase (n = 26)	Total (N = 65)	Chi-square (p-value)
History suggestive of SDB	28/41 (68.3%)	13/41 (31.7%)	41 (63.1%)	p = 0.074
No history suggestive of SDB	11/24 (45.8%)	13/24 (54.2%)	24 (36.9%)	

Admissions compared to the UK

Our total admissions were 39 (60%), and 26 were daycase (40%). When the UK guidelines were applied, the proposed admissions became 31 (47.7%) and the daycase went up to 34 (52.3%). Out of the 34 patients who were eligible for daycase surgery based on British guidelines, 19 (55.9%) were successfully discharged on the same day, while 15 (44.1%) required overnight admission. The decision to admit these patients was influenced by additional considerations, such as a documented history of sleep apnea and obesity, although not exceeding the >98th percentile threshold defined by the British guidelines. Four patients were admitted despite no clear documentation of the reason or for social reasons. A summary of the frequency of each reason is presented in Table 4.

**Table 4:** Reasons for admission Number of patients is presented as (n), and percentages are shown in parentheses (%), calculated from the total number of patients in this group (15 patients).

Admission rationale in patients eligible for daycase tonsillectomy	Number of patients (n, %)
Documented sleep apnea	9 (60%)
History of sleep apnea + obesity (> 95th and <98th percentile)	2 (13.3%)
Unknown reason/social reasons	4 (26.7%)

Postoperative complications

Out of all cases (n = 65), six (9.2%) patients had a documented postoperative complication. Three patients (4.6%) had postoperative pain and dysphagia that required re-admission for pain control. While the other three (4.6%) presented in the postoperative period with a post-tonsillectomy bleed. Four out of all postoperative complications (N = 6) occurred in the daycase group (66.7%), including the three patients who required re-admission due to postoperative pain and one patient with a post-tonsillectomy bleed.

## Discussion

This study evaluated the feasibility of performing daycase pediatric adenotonsillectomy at our tertiary care center. Our findings showed that a higher proportion of patients (n = 34, 52.3% vs. n = 26, 40%) would have been eligible for daycase surgery when applying the UK GIRFT criteria. Despite applying the British guidelines to our population retrospectively, the presumed daycase rates remained lower than the recommended 80% target [3].

Among those who met the GIRFT discharge criteria but were still admitted (n = 15, 44.1%), the majority (n = 9, 60%) had a documented history of SDB with witnessed episodes of apnea. There is no consensus regarding the postoperative course for patients with obstructive sleep apnea (OSA) undergoing tonsillectomy with or without adenoidectomy. Prospective data on same-day discharge criteria and the systemic examination of possible postoperative complications in children with OSA remain limited [2].

A review of the literature shows significant variation in the postoperative course of this group of patients among different pediatric hospitals [1]. Multiple clinical practice guidelines were published for these cases, including those released by the American Academy of Otolaryngology-Head and Neck Surgery (AAO-HNS), the American Academy of Pediatrics (AAP), and the American Society of Anesthesiologists (ASA). The AAP guidelines published in 2012, advised overnight admission for children with an apnea-hypopnea index (AHI) of ≥ 24 and a preoperative oxygen saturation (SaO₂) nadir below 80% on a preoperative polysomnography. However, the ASA guidelines published in 2013 recommended hospital admission for severe OSA patients with AHI ≥ 10. Later, the AAO-HNS committee released its clinical guidelines in 2019 that considered the more conservative AHI index ≥ 10 and a pre-operative oxygen saturation of < 80% as a cutoff for postoperative overnight admission in OSA patients [1].

Practical application of any of these guidelines is difficult, as we relied on clinical diagnosis rather than polysomnography for most patients. In our study population, only three out of 41 patients (7.3%) with a history suggestive of SDB and witnessed apneas had a preoperative polysomnography. Hanss et al. looked at 96 pediatric patients with clinically diagnosed OSA who were managed as daycases in a French tertiary care hospital in 2011. None of the patients had a respiratory-related postoperative complication [4]. Baguley et al. [5] explored the risk of postoperative respiratory complications (classified into major respiratory complications, desaturation, and laryngospasm) in pediatric patients with mild to moderate OSA who were over three years of age (a total of 100 cases). The study showed that no major respiratory complications were reported. Only one child required supplemental oxygen in recovery, and one child experienced laryngospasm. Three patients required supplemental oxygen on the ward beyond the immediate six hours postoperative period. They concluded that patients with mild to moderate OSA, who are otherwise healthy and over three years of age, can be safely discharged after completing six hours of postoperative monitoring [5].

Given the lack of local data on the severity of sleep apnea in our population and the potential for postoperative complications, a more conservative approach was taken, with a lower threshold to admit OSA patients. Only 13 out of 41 (31.7%) OSA patients were managed as daycases in our study, while the remaining 28 (68.3%) were admitted for observation. None of the 41 patients experienced any major postoperative respiratory complications. Although data on pediatric OSA severity in the UAE are very limited, our study suggests that more lenient daycase criteria could be safely applied to a select group of patients. Additionally, local risk assessment tools should be developed to help guide decisions on when to refer patients for a preoperative polysomnography.

Our study group showed that 16.9% of all operated children had a medical comorbidity, and all of them were admitted for overnight observation. The most common comorbidity was asthma, followed by syndromic conditions and panhypopituitarism, which were equally represented. The GIRFT guidelines list the following comorbidities as relative risk factors for daycase surgery: Down syndrome, achondroplasia, severe cerebral palsy, significant respiratory disease, craniofacial abnormalities, congenital heart disease, hematological or clotting disorders, neuromuscular disorders, and co-existing upper airway abnormalities [3]. Some medical conditions seen in our study population were not mentioned in the GIRFT criteria, such as autism, ADHD, and panhypopituitarism. A study published in 2016 found that ADHD increased the risk of post-tonsillectomy bleeding by approximately 8.7 times [6]. Panhypopituitarism, on the other hand, remains an under-recognized condition with very limited evidence on postoperative management following adenotonsillectomy. However, general literature highlights it as a risk factor for metabolic and hemodynamic instability during the perioperative period [7]. 

Postoperative complication was reported in six out of all 65 patients, three of whom had poor oral intake and dehydration, and the other three had post-tonsillectomy bleeding (4.6%). One case was recorded in a daycase patient, while the other two had been admitted overnight after surgery. A Jordanian study that was published in 2019 demonstrated no difference in the reported post-tonsillectomy hemorrhage between admission and daycase groups [8].

GIRFT guidelines recommended overnight admission for children with BMI > 98th centile and children with body weight < 14 kg [3]. According to the Centers for Disease Control (CDC) and Prevention, obesity is defined as a BMI-for-age ≥ 95th percentile in children two to 19 years of age. Studies that explored obesity-related postoperative complications used the same cut-off of ≥ 95th percentile as an inclusion criterion [9]. Having a lower cut-off is expected to result in higher admission rates. 

Study limitations

Data were extracted retrospectively from a medical record system, which can be influenced by the accuracy and completeness of the physician’s documentation. As noted in our chart review, not all medical records documented the living distance from the hospital. Additionally, the surgical technique was not analyzed in this study. Analyzing the surgical techniques used would be of interest, as they may influence postoperative pain and same-day discharge eligibility.

## Conclusions

Our retrospective chart review showed that daycase tonsillectomy and adenotonsillectomy appear to be a safe approach for a substantial portion of our pediatric population, even in a tertiary care hospital that tends to receive more complex referrals. However, the proposed target by the GIRFT study of an 80% daycase rate is difficult to implement, considering the complexity of our patient population. To help improve our discharge rates and get closer to the internationally suggested target, tailored institution-specific discharge protocols need to be developed. In addition, clear preoperative risk assessment tools for patients with SDB and OSA are needed. Auditing our existing discharge criteria in a way that balances safety, efficiency, and resource use is crucial. 
